# Internet Addictive Individuals Share Impulsivity and Executive Dysfunction with Alcohol-Dependent Patients

**DOI:** 10.3389/fnbeh.2014.00288

**Published:** 2014-08-25

**Authors:** Zhenhe Zhou, Hongmei Zhu, Cui Li, Jun Wang

**Affiliations:** ^1^Department of Psychiatry, Wuxi Mental Health Center, Wuxi, China

**Keywords:** Internet addiction disorder, alcohol dependence, impulsivity, executive function, working memory

## Abstract

Internet addiction disorder (IAD) should belong to a kind of behavioral addiction. Previous studies indicated that there are many similarities in the neurobiology of behavior and substance addictions. Up to date, although individuals with IAD have difficulty in suppressing their excessive online behaviors in real life, little is known about the patho-physiological and cognitive mechanisms responsible for IAD. Neuropsychological test studies have contributed significantly to our understanding of the effect of IAD on the cognitive function. The purpose of the present study was to examine whether Internet addictive individuals share impulsivity and executive dysfunction with alcohol-dependent individuals. Participants include 22 Internet addictive individuals, 22 patients with alcohol dependence (AD), and 22 normal controls (NC). All participants were measured with BIS-11, go/no-go task, Wisconsin Card Sorting Test, and Digit span task under the same experimental condition. Results showed that Barratt impulsiveness scale 11 scores, false alarm rate, the total response errors, perseverative errors, failure to maintain set of IAD and AD group were significantly higher than that of NC group, and hit rate, percentage of conceptual level responses, the number of categories completed, forwards scores, and backwards scores of IAD and AD group were significantly lower than that of NC group, however, no differences in above variables between IAD group and AD group were observed. These results revealed that the existence of impulsivity, deficiencies in executive function and working memory in an IAD and an AD sample, namely, Internet addictive individuals share impulsivity and executive dysfunction with alcohol-dependent patients.

## Introduction

Internet addiction disorder (IAD) originates from the phenomenon of the Internet now being a part of the common person’s daily life. It is well known that the Internet provides persons with the abilities to easily acquire information, learn new knowledge, gain and maintain relationships, and even make money. In short, the Internet has been instrumental in improving people quality of life. IAD is defined as a person’s inability to control his or her use of the Internet, which eventually leads to psychological, social, school, and work difficulties or dysfunction in an individual’s life (Young and Rogers, 1998; Davis, 2001). Because IAD is recognized internationally and is known to be linked with academic and social dysfunction, it has been increasingly recognized as a mental disorder. Recent investigations of its high prevalence in youth populations, combined with evidence that IAD is a maladaptive behavior with potentially serious occupational and mental health consequences, support the validity of the diagnosis (Ko et al., 2012). A previous study that investigated deficient inhibitory control in persons with IAD using a go/no-go task by event-related potentials (ERPs) indicated adult individuals with IAD were more impulsive than controls and shared neuropsychological and ERPs characteristics of compulsive–impulsive spectrum disorder (Zhou et al., 2010). Another study using the cue-related go/no-go switching task showed that individuals with IAD present cognitive biases toward information related to Internet gaming and poor executive functioning skills (lower mental flexibility as well as response inhibition) (Zhou et al., 2012). Impairments in executive functioning, including response monitoring, have been suggested as a hallmark feature of impulse control disorders. The error-related negativity (ERN) reflects person’s ability to monitor behavior. A recent study examines whether individuals with IAD display response monitoring functional deficit characteristics in a modified Eriksen flanker task (Zhou et al., 2013). In the study, subjects and controls completed the modified Eriksen flanker task while measured with ERPs. Results showed that the mean ERN amplitudes of total error response conditions at frontal electrode and central electrode sites of subjects were reduced compared with controls. These results indicated that individuals with IAD display response monitoring functional deficit characteristics and share ERN characteristics of individuals with compulsive–impulsive spectrum disorder. Subtypes of IAD include excessive gaming, sexual preoccupations, and e-mail/text messaging. Three subtypes share the common components, i.e., preoccupation, mood modification, unplanned use, withdrawal, tolerance, and functional impairment (Block, 2008). By using the Diagnostic and Statistical Manual of Mental Disorders (Fourth Edition, DSM-IV) criteria, some scholars suggest that IAD is an impulse disorder or at least related to impulse control disorder (Beard and Wolf, 2001; Shaw and Black, 2008).

Behavioral addiction is a form of addiction not caused by the usage of drugs. It consists of a compulsion to repeatedly engage in an action until it causes negative consequences to the person’s physical, mental, and social well-being. Behavior persisting in spite of these consequences can be taken as a sign of addiction (Potenza, 2006; Parashar and Varma, 2007). According to above interpretation, IAD should belong to a kind of behavioral addiction. The drug based reinforcement and reward based learning processes are the most important mechanism of addictions. Impulsivity is considered as the tendency to act prematurely without foresight (Dalley et al., 2011). According to both animal and human studies, there are two forms of impulsivity: one depends on the temporal discounting of reward; another on motor or response disinhibition (Buckholtz et al., 2010). Barratt impulsiveness scale 11 (BIS-11) is considered more of a trait measure of impulsivity (Patton and Stanford, 1995). The go/no-go task is used for operational measures of impulsivity. Studies displayed that impulsivity is commonly associated with addiction to drugs from different pharmacological classes (Dick et al., 2010; Ersche et al., 2011; Molander et al., 2011; Economidou et al., 2012).

Executive function and working memory are critical features of cognition. The Wisconsin Card Sorting Test (WCST) is a neuropsychological test of “set-shifting,” i.e., the ability to display flexibility in the face of changing schedules of reinforcement (Monchi et al., 2001). WCST is employed to assess the “frontal” lobe functions including strategic planning, organized searching, utilizing environmental feedback to shift cognitive sets, directing behavior toward achieving a goal, and modulating impulsive responding. Because of its reported sensitivity to frontal lobe dysfunction, WCST has been considered a measure of executive function. Individuals with substance dependence present working memory impairments as well as executive dysfunctions, which include reasoning, problem solving, inhibitory controlling, and decision-making (Crean et al., 2011; Hanson et al., 2011; Kiluk et al., 2011; Thoma et al., 2011; Yücel et al., 2012). Working memory refers to a brain system that provides temporary storage and manipulation of the information necessary for such complex cognitive tasks as language comprehension, learning, and reasoning. Digit span task (forwards/backwards) from the Wechsler Adult Intelligence Scale was used to index the maintenance and manipulation of verbal information in working memory (Baddeley, 1992).

Many studies displayed that individual with IAD present executive dysfunctions and reward/punishment sensitivities. For example, a study, which used a gambling task to simulate extreme win/lose situations to find the reward/punishment sensitivities after continuous wins and losses, showed that higher superior frontal gyrus activations after continuous wins for IAD subjects than for normal controls (NC). The brain activities in IAD subjects were not disturbed by their losses. In addition, IAD participants showed decreased posterior cingulate activation compared to NC after continuous losses. These results indicated that IAD subjects showed enhanced sensitivity to win and decreased sensitivity to lose (Dong et al., 2013a). Studies on neuroimaging indicated that individuals with IAD present executive dysfunctions including attentional selections and decision-making (Sun et al., 2009; Pawlikowski and Brand, 2011; Dong et al., 2013b).

Diminished control is a core defining concept of substance dependence or addiction. The concept of behavioral addictions has some scientific and clinical heuristic value, but remains controversial. Several behavioral addictions, such as pathological gambling, pathological kleptomania, and pathological shopping, have been hypothesized as having similarities to substance addictions. Additionally, these behavioral addictions are classified as impulse control disorders, a separate category from substance use disorders. However, not all impulse control disorders should be considered behavioral addictions (Grant et al., 2010). For example, intermittent explosive disorder is a behavioral disorder characterized by extreme expressions of anger, often to the point of uncontrollable rage, that are disproportionate to the situation at hand. Impulsive aggression is unpremeditated, and is defined by a disproportionate reaction to any provocation, real or perceived. Intermittent explosive disorder does not share characters of behavioral addictions. Previous studies indicated that there are many similarities in the neurobiology of behavior and substance addictions (Leeman and Potenza, 2012). Behavioral and substance addictions have many similarities in natural history, adverse consequences, and phenomenology. Individuals with behavioral addictions and those with substance use disorders both score high on self-report measures of impulsivity and sensation-seeking and generally low on measures of harm avoidance (Lejoyeux et al., 1997; Kim and Grant, 2001; Grant and Kim, 2002). Prevalence studies showed that individuals with IAD or substance dependence display common characteristics including high novelty-seeking behavior and low reward dependence (Ko et al., 2012). Adolescents with alcohol dependence were more likely to have IAD and show certain psychosocial characters including high behavior activation, low self-esteem, low family function, and life satisfaction (Ko et al., 2008). Substance dependence has been associated with sensation-seeking (Sargent et al., 2010), which has also been positively correlated with IAD (Chiu et al., 2004; Mehroof and Griffiths, 2010).

In research work, Internet Addiction Test (IAT, Young, 1999), Diagnostical Questionnaire (DQ, Young, 1996), the modified Diagnostic Questionnaire for Internet Addiction (YDQ, Beard and Wolf, 2001), and the Compulsive Internet Use Scale (CIUS, Meerkerk et al., 2009) are usually used as diagnosis instruments. It is becoming a common opinion that the IAT is not completely reliable and valid psychometric instrument nowadays. DQ is a significant contribution in providing a concrete basis for establishing problematic Internet use. However, there is limited research on Internet addiction including a representative sample to use as a comparison for those being diagnosed. As a result, no reliable and valid diagnostic criteria have been determined. Although YDQ does not solve all of the previously mentioned problems, it may help strengthen Young’s proposed criteria (Beard and Wolf, 2001). CIUS showed good factorial stability across time and across different samples and subsamples. The internal consistency is high, and high correlations with concurrent and criterion variables demonstrate good validity (Meerkerk et al., 2009).

Being a non-invasive method, neuroimaging plays important roles in the investigation of neurobiological mechanism and adequate treatments of IAD and drug abuse. Until now, there are several neuroimaging studies on IAD. Studies indicated that individuals with IAD shared impulsivity features of individuals with substance dependence (Dong et al., 2011, 2012, 2014; Yuan et al., 2011; Zhou et al., 2013).

Up to date, although individuals with IAD have difficulty suppressing their excessive online behaviors in real life, little is known about the patho-physiological and cognitive mechanisms responsible for IAD (Weinstein and Lejoyeux, 2010). Neuropsychological test studies have contributed significantly to our understanding of the effect of IAD on the cognitive function. Under the same experimental condition to assess impulsivity and executive function of IAD and substance dependence (such as alcohol dependence) may not only help guide decisions as to whether or not IAD should be grouped together with substance use disorders, but also play important roles in the investigation of neurobiological mechanism and adequate treatments of IAD. In this study, participants are individuals with IAD, patients with alcohol dependence (AD) and NC. All participants were measured with BIS-11, go/no-go task, WCST, and Digit span task under the same experimental condition. The purpose of the present study was to examine whether Internet addictive individuals share impulsivity and executive dysfunction with alcohol-dependent individuals.

## Materials and Methods

### Time and setting

The experiment was completed in the Department of Psychology and the Department of Psychiatry at Wuxi Mental Health Center, China, from May 2011 to October 2013.

### Diagnostic approaches and participants

#### IAD group

The diagnostic criteria of IAD group included: (i) met the criteria of the modified Diagnostic Questionnaire for Internet Addiction (YDQ) (Beard and Wolf, 2001), i.e., individuals who answered “yes” to questions one through five and at least any one of the remaining three questions were classified as suffering from IAD; (ii) whose age were more than 18 years old; (iii) did not meet criteria of any DSM-IV axis I disorder or personality disorders by administering a structured clinical interview (Chinese version); (iv) were not smokers; and (v) had not a diagnosis of alcohol or substance dependence, neurological disorders, all kinds of head injury, or systemic disease that might affect the central nervous system. The duration of the disorder was confirmed via a retrospective diagnosis. Subjects were asked to recall their life-style when they were initially addicted to the Internet. In order to confirm that they were suffering from Internet addiction, we retested them with the criteria of the modified YDQ. The reliability of these self-reports from the IAD subjects were confirmed by talking with their parents via telephone. The IAD subjects spent 11.20 ± 1.81 h/day on online activities (including gaming, Internet shopping, pornography, Internet social interaction, virtual society, and obtaining information). The days of Internet use per week was 6.41 ± 0.6. We verified this information from the roommates and co-workers of the IAD subjects that they often insisted being on the Internet late at night, disrupting others’ lives despite the consequences. IAD group was recruited from Psychology Department of Wuxi Mental Health Center. They have regulated sleep patterns and did not ingest large quantities of caffeinated and energetic drinks by medical staffs’ management. Twenty-two subjects were recruited as IAD group.

#### AD group

The diagnostic criteria of AD group included: (i) met the criteria of DSM-IV for alcohol dependence; (ii) no medication was received before 2 weeks; (iii) were not smokers; and (iv) had not a diagnosis of comorbid psychiatric illness (with the exception of depression in the alcohol-dependent group), history of head injury or neurological disorder. Alcohol-dependent subjects were in-patients at Psychiatry Department of Wuxi Mental Health Center. Sobriety at time of testing was confirmed by breath alcohol readings ≤0.01 mg/l. All subjects were abstinent for >1 week. The mean duration of abstinence was 15 days.

#### NC group

The controls were selected from citizens lived in Wuxi city, Jiangsu Province, China through local advertisement. Controls were excluded from the study if they were smokers; or had a diagnosis of alcohol or substance dependence, neurological disorders, all kinds of head injury, or systemic disease that might affect the central nervous system. Twenty-two healthy persons were recruited as NC group. Referred from a previous IAD study (Ko et al., 2009a), we chose NC who spent <2 h/day on the Internet. The NC were tested with the YDQ criteria modified by Beard and Wolf to certificate they were not suffering from IAD. All participants were Chinese.

All participants underwent a clinical assessment by a psychiatric residency to collect information on medication, socio-demographic data, and to confirm/exclude an IAD and AD diagnosis. In this study, we gave all participants a written informed consent to participate and all were paid. The protocol for the research project was approved by the Ethics Committee of Wuxi Mental Health Center, China.

The demographic characteristics of the sample are detailed in Table 1.

**Table 1:** **Demographic characteristics and clinical data of the sample**.

	IAD	AD	NC	Test statistic	*Post hoc* analyses
Sex ratio (M/F)	22 (16/6)	22 (18/4)	22 (15/7)	χ^2^ = 5.64, *P* = 0.12, NS	–
Mean age (SD)	28 (7)	30 (6)	28 (7)	*F*(2,66) = 2.32, NS	–
Age range	21–36	23–38	21–36	–	–
Education (SD)	9 (3)	9 (3)	9 (3)	*F*(2,66) = 3.21, NS	–
Years of addiction (SD)	4 (3)	3 (3)	–	*t* = 1.355, *P* = 0.62, NS	–
SADQ (SD)	–	32.2 (17.3)	–	–	–
HAMD (SD)	9.1 (3.1)	8.9 (3.5)	5.1 (3.6)	*F*(2,65) = 5.88, *P* = 0.23, NS	–

*IAD, internet addictive individual group; AD, alcohol-dependent individual group; NC, normal control group; M, male; F, female; SD, standard deviation; SADQ, severity of alcohol dependence questionnaire; HAMD, Hamilton depression scale; NS, not significant*.

### Tasks and procedure

All participants completed the Hamilton Depression Scale (HAMD) (version of 17 items) (Hamilton, 1967) to measure depressive symptoms and BIS-11 to measure impulsivity. BIS-11 is a questionnaire on which participants rate their frequency of several common impulsive or non-impulsive behaviors/traits on a scale from 1 (rarely/never) to 4 (almost always/always). BIS-11 includes 30 items and is divided into three subscales including attentional key, motor key, and non-planning key, to determine overall impulsiveness scores, all items are summed, with higher scores indicating greater impulsivity. The AD group completed the Severity of Alcohol Dependence Questionnaire (SADQ) (Stockwell et al., 1983).

The Neuropsychological tests included the following measures.

#### Go/no-go task

E-Prime software 2.0 (Psychology Software Tools Inc., Sharpsburg, NC, USA) was used for the go/no-go task. The task, referred from pervious study (Zhou et al., 2010), involved the serial presentation on a computer screen of eight different two-digit numerical stimuli (four go stimuli and four no-go stimuli), displayed white on black background (1.5 cm × 1.5 cm in size). A total of 160 stimuli were presented in 20 blocks. Each block included eight trials, and pseudo-randomly presented with no more than three consecutive trials with either a go or no-go stimulus so that withholding a response involved overcoming an established response tendency. The go stimuli in any blocks were “08,” “63,” “74,” and “25”; the no-go were “58,” “19,” “14,” and “79.” Subjects were told that the task involved learning when to go (bar press as quickly as possible) or not to go (withhold response) and that responses after some numbers would result in winning money ($0.16 per trial) but responses after others would result in losing money ($0.16 per response). The response window was 1000 ms and the inter-trial interval (ITI) was 1500 ms. Reward contingencies (green background with +$0.16 in white) or punishment contingencies (red background with −$0.16 in white) were presented on the computer screen for 1000 ms immediately after a response (within the 1500 ms ITI). The experiment included a practice phase and a recording phase. The practice phase consisted of 16 go and no-go trials. The percentage of hits and reactive time (RT) to go stimuli and the percentage of false alarms to no-go stimuli were used for analysis. When the button was pressed within 200–1000 ms after the presentation of a go stimulus, the response was confirmed as correct. Lack of a response in this latency window was defined as a miss, whereas responses made within this window to no-go stimuli were defined as false alarms. False alarms were defined for each modality separately. The percentage of correct responses to go stimuli was confirmed as 100 × N (target detections) divided by the total number of go stimuli. The percentage of false alarms to no-go stimuli was confirmed as 100 × N divided by the sum of no-go stimuli presented. RT was measured from the onset of the go stimulus to the button press.

#### Wisconsin card sorting test

The WCST (Beijing Ka Yip Wise Development Co., Ltd, computerized version VI) was present graphically on a computer screen. The WCST entailed matching stimulus cards with one of four category cards, in which the stimuli were multidimensional according to color, shape, and number, each dimension determining a sorting rule. By trial and error, the participant has to decide a preordained sorting rule given just the feedback (“Right” or “Wrong”) on the screen after each sort. After 10 consecutive correct sorts the rule changed. There were up to six attempts to derive a rule, providing five rule shifts in the following sequence (color – shape – number – color – shape number), with each rule attainment referred to as “completing a category.” Participants were not informed of the correct sorting principle and that the sorting principal shifts during the measurement; measuring continues until all 128 cards were sorted and irrespective of whether the participant achieved completes all the rule shifts. Two types of errors were possible, perseverative errors, in which the participant made a response in which they persist with a wrong sorting rule, and non-perseverative errors. In this study, five main types of WSCT were used for analysis: (i) the total response errors; (ii) perseverative errors; (iii) percentage of conceptual level responses; (iv) the number of categories completed; and (v) failure to maintain set.

#### Digit span task

Wechsler Adult Intelligence Scale-Revised China (WAIS-RC, Beijing Ka Yip Wise Development Co., Ltd, computerized version) was used for measurement of Digit span task. All participants are given sets of digits to repeat initially forwards then backwards. This is a test of immediate auditory recall and freedom from distraction. The participant was told to listen carefully because he or she will say a series of numbers and ask him or her to repeat them back in the same order. The first series is three numbers, such as “3, 9, 2.” Each number is said in a monotone voice, one second apart. The person repeats those numbers back. The next step is to speak a series of four numbers, such as, “4, 7, 3, 1.” Again, the individual repeats those back. Continue in the same manner by increasing the series of numbers to five and asking the participant to repeat the numbers back.

### Statistical analysis

Data were analyzed using SPSS (SPSS, Chicago, IL, USA). Sex ratio among IAD group, AD group, and NC group were analyzed with χ^2^ tests. Comparisons of years of addiction between IAD group and AD group were done using independent-sample *t*-tests. Comparisons of HAMD scores, BIS-11 scores, data of go/no-go task, WSCT, and Digit span task among IAD group, AD group, and NC group were done using one-way analysis of variance (ANOVA). Least square difference (LSD) tests were performed as *post hoc* analyses if indicated. Alpha values of 0.05 were considered significant throughout.

## Results

### Comparisons of BIS-11 scores among IAD group, AD group, and NC group

Using attentional key scores, motor key scores, non-planning key scores, and BIS-11 total scores as dependent variable, respectively, a one-way ANOVA revealed a significant main effect of Group (IAD group, AD group, and NC group). *Post hoc* LSD tests showed that attentional key scores, motor key scores, non-planning key scores, and BIS-11 total scores of IAD and AD group were significantly higher than that of NC group (for attentional key scores, *p * = 0.038 and 0.028, respectively; for motor key scores, *p * = 0.030 and 0.036, respectively; for non-planning key scores, *p * = 0.017 and 0.049, respectively; for BIS-11 total scores, *p * = 0.022 and 0.035, respectively), while above four main type data were not significantly different between IAD and AD group (all *p* > 0.05) (Table 2).

**Table 2:** **BIS-11 scores [mean (SD)] in IAD group (*n* = 22), AD group (*n* = 22), and NC group (*n* = 22)**.

Variable	IAD	AD	NC	Test statistic
Attentional key	32.5 (2.6)	33.0 (3.7)	29.6 (2.8)	*F*(2,65) = 10.98, *P* = 0.012, Cohen’s *d* = 0.98
Motor key	23.3 (3.1)	23.9 (4.2)	21.4 (3.9)	*F*(2,65) = 12.57, *P* = 0.020, Cohen’s *d* = 0.76
Non-planning key	22.0 (3.1)	22.7 (1.1)	20.8 (3.1)	*F*(2,65) = 9.67, *P* = 0.038, Cohen’s *d* = 0.62
Total scores	77.5 (8.1)	73.8 (9.2)	71.2 (7.5)	*F*(2,65) = 62.50, *P* = 0.001, Cohen’s *d* = 1.18

*IAD, internet addictive individual group; AD, alcohol-dependent individual group; NC, normal control group; SD, standard deviation*.

### Comparisons of RTs, hit rate, and false alarm rate among IAD group, AD group, and NC group

Using RTs as dependent variable, a one-way ANOVA revealed no main effect of Group (IAD group, AD group, and NC group). Using hit rate and false alarm rate as dependent variable, respectively, a one-way ANOVA revealed a significant main effect of Group (IAD group, AD group, and NC group). *Post hoc* LSD tests showed that false alarm rate of IAD and AD group were significantly higher than that of NC group, and hit rate was significantly lower than that of NC group (for false alarm rate, *p * = 0.027 and 0.034, respectively; for hit rate, *p * = 0.017 and 0.020, respectively), while false alarm rate and hit rate was not significantly different between IAD and AD group (all *p* > 0.05) (Table 3).

**Table 3:** **RTs, hit rate, and false alarm rate [mean (SD)] in IAD group (*n* = 22), AD group (*n* = 22), and NC group (*n* = 22)**.

Variable	IAD	AD	NC	Test statistic
RT (ms)	541 (59)	542 (63)	539 (68)	*F*(2,65) = 4.37, *P* = 0.13, Cohen’s *d* = 0.00
Hit rate	0.902 (0.003)	0.903 (0.002)	0.915 (0.002)	*F*(2,65) = 15.61, *P* = 0.019, Cohen’s *d* = 0.89
False alarm rate	0.043 (0.057)	0.042 (0.058)	0.016 (0.015)	*F*(2,65) = 13.25, *P* = 0.023, Cohen’s *d* = 0.81

*IAD, internet addictive individual group; AD, alcohol-dependent individual group; NC, normal control group; SD, standard deviation*.

### Comparisons of WSCT data among IAD group, AD group, and NC group

Using total response errors, perseverative errors, percentage of conceptual level responses, the number of categories completed, and failure to maintain set as dependent variable respectively, a one-way ANOVA revealed a significant main effect of Group (IAD group, AD group, and NC group). *Post hoc* LSD tests showed that total response errors, perseverative errors, and failure to maintain set of IAD and AD group were significantly higher than that of NC group, and percentage of conceptual level responses and the number of categories completed of IAD and AD group were significantly lower than that of NC group (for total response errors, *p * = 0.041 and 0.022, respectively; for perseverative errors, *p * = 0.039 and 0.040, respectively; for failure to maintain set, *p * = 0.024 and 0.027, respectively; for percentage of conceptual level responses, *p * = 0.011 and 0.021, respectively; for the number of categories completed, *p * = 0.043 and 0.0391, respectively), while above five main type data were not significantly different between IAD and AD group (all *p* > 0.05) (Table 4).

**Table 4:** **WSCT data [mean (SD)] in IAD group (*n* = 22), AD group (*n* = 22), and NC group (*n* = 22)**.

Variable	IAD	AD	NC	Test statistic
Total response errors (%)	35.8 (20.6)	36.1 (23.2)	19.6 (21.8)	*F*(2,65) = 21.28, *P* = 0.001, Cohen’s *d* = 1.43
Perseverative errors (%)	21.3 (12.1)	22.9 (14.2)	13.4 (13.0)	*F*(2,65) = 18.55, *P* = 0.001, Cohen’s *d* = 1.27
Conceptual level responses (%)	53.0 (6.1)	54.7 (7.2)	60.8 (8.0)	*F*(2,65) = 5.77, *P* = 0.040, Cohen’s *d* = 0.56
Number of categories completed	5.7 (2.1)	5.8 (3.3)	6.5 (3.5)	*F*(2,65) = 10.50, *P* = 0.001, Cohen’s *d* = 1.09
Failure to maintain set	0.8 (0.6)	0.8 (1.2)	0.3 (0.5)	*F*(2,65) = 3.50, *P* = 0.011, Cohen’s *d* = 0.99

*IAD, internet addictive individual group; AD, alcohol-dependent individual group; NC, normal control group; SD, standard deviation*.

### Comparisons of digit span task scores among IAD group, AD group, and NC group

Using forwards scores and backwards scores as dependent variable, respectively, a one-way ANOVA revealed a significant main effect of Group (IAD group, AD group, and NC group). *Post hoc* LSD tests showed that forwards scores and backwards scores of IAD and AD group were significantly lower than that of NC group (for forwards scores, *p * = 0.016 and 0.025, respectively; for backwards scores, *p * = 0.017 and 0.041, respectively), while above two main type data were not significantly different between IAD and AD group (all *p* > 0.05) (Table 5).

**Table 5:** **Digit span scores [mean (SD)] in IAD group (*n* = 22), AD group (*n* = 22), and NC group (*n* = 22)**.

Variable	IAD	AD	NC	Test statistic
Forwards scores	8.5 (1.6)	8.1 (1.2)	11.6 (1.5)	*F*(2,65) = 8.28, *P* = 0.014, Cohen’s *d* = 0.86
Backwards scores	8.3 (3.8)	7.9 (4.2)	10.1 (3.0)	*F*(2,65) = 4.59, *P* = 0.026, Cohen’s *d* = 0.59

*IAD, internet addictive individual group; AD, alcohol-dependent individual group; NC, Normal control group; SD, standard deviation*.

## Discussion

This study is the first to test impulsivity, executive function, and working memory between Internet addictive individuals and with alcohol-dependent patients under the same experimental condition. In this study, impulsivity was measured with BIS-11 and a go/no-go task, executive function was assessed with WCST and working memory was tested with Digit span task. Our results indicate the existence of impulsivity in an IAD group and an AD group, deficiencies in executive function and working memory in an IAD and an AD sample.

Internet addiction disorder and alcohol dependence involve continued use of alcohol and Internet, respectively despite negative consequences, i.e., loss of behavioral control of alcohol and Internet use. Impulsivity refers to premature, unduly risky, and poorly conceived actions. Dysfunctional impulsivity includes deficits in attention, lack of reflection, or insensitivity to consequences, all of which may occur in addiction (Evenden, 1999; de Wit, 2009).

A recent study using traditional neuropsychological tests including the Stroop and computerized neuropsychological tests showed that IAD group exhibited more trait impulsivity than the healthy control group, Furthermore, IAD group performed more poorly than the healthy control group in a computerized stop signal test, and no group differences appeared for other neuropsychological tests, which indicated that individuals with IAD exhibited impulsivity as a core personality trait and in their neuropsychological functioning (Choi et al., 2014). Many studies displayed that alcohol-dependent patients present neurocognitive deficits in memory, learning, visuospatial functions, psychomotor speed processing, executive functions and decision-making, and the cognitive alterations are directly related to compliance with treatment and maintenance of withdrawal (Parsons, 1998). In our study, there were significant differences in BIS-11 scores among IAD group, AD group, and NC group; however, no differences in BIS-11 scores between IAD group and AD group were observed. Simultaneously, in go/no-go task, there were significant differences in false alarm rate and hit rate among IAD group, AD group, and NC group, and no differences in false alarm rate and hit rate between IAD group and AD group were observed. Above two tests indicate that both IAD and AD are more impulsive than controls, and Internet addictive individuals share impulsivity with alcohol-dependent patients.

Executive functions include abstract thinking, motivation, decision-making, planning, attention to tasks, and inhibition of impulsive responses. Although WCST exists some acknowledged weaknesses in interpretation of the profiles, i.e., difficulties in task performance could be caused by set-shifting, poor abstraction and conceptualization, or attentional problems, this procedure integrates multiple measurements of executive processes and is the most widely reported neuropsychological task. WCST commonly was used for neuropsychological measure of cognitive flexibility (or set-shifting ability). Our study results displayed that the total response errors, perseverative errors, and failure to maintain set of IAD and AD group were significantly higher than that of NC group, while above three main type data were not significantly different between IAD and AD group. Additionally, percentage of conceptual level responses and the number of categories completed of IAD and AD group were significantly lower than that of NC group, while above two main type data were not significantly different between IAD and AD group. These results indicate that both Internet addictive individuals and alcohol-dependent patients present the same property of executive dysfunctions. Many previous neuropsychological researches indicated that Internet-related cues interfere with control processes mediated by the prefrontal cortex and prefrontal brain areas, and Internet-related stimuli interfere with decision-making and other prefrontal functions, such as working memory and further executive functions (Brand et al., 2014). Our results support that the reductions of prefrontal control processes play a major role in developing and maintaining an addictive use of the Internet.

Working memory is the system that actively holds multiple pieces of transitory information in the mind, where they can be manipulated. Working memory is generally used synonymously with short-term memory, and it depends on how the two forms of memory are defined (Cowan, 2008). The cognitive processes needed to achieve this include the executive and attention control of short-term memory, which permit interim integration, processing, disposal, and retrieval of information (Rouder et al., 2011). This study results showed that by measurement of Digit span task, there were significant differences in forwards scores and backwards scores among IAD group, AD group, and NC group. Forwards scores and backwards scores of IAD and AD group were significantly lower than that of NC group, however, forwards scores and backwards scores were not significantly different between IAD and AD group. These results manifest that Internet addictive individuals share impairment of working memory with alcohol-dependent patients.

In conclusion, the results of this study clearly show that the existence of impulsivity, deficiencies in executive function, and working memory in an IAD and an AD sample, namely, Internet addictive individuals share impulsivity and executive dysfunction with alcohol-dependent individuals. Understanding the biological effects and characters of cognitive function of IAD on the human brain may provide insight into the pathogenesis of IAD and treatment. Up to date, although there is much argument on the diagnostic definition of IAD, numerous neuroimaging studies had highlighted structural and functional abnormalities in individuals with IAD similar to other type of addictive disorders, such as substance addiction and behavioral addiction (Fischl and Dale, 2000; Ko et al., 2009b). Our study using neuropsychological test proved that cognitive dysfunction in individuals with IAD similar to alcohol-dependent individuals. Neurocognitive assessment may be a useful tool for the detection and assessment of the progress of these alterations, as well as for the cognitive rehabilitation and psychosocial reinsertion of individuals with IAD.

A limitation of this study is that this study used the modified Diagnostic Questionnaire for Internet Addiction scores of higher than six as an indicator of IAD. Although this questionnaire is a frequently used instrument for assessing IAD, its validity as a diagnostic instrument has been questioned (Beard, 2005). Future studies may utilize other measures of assessing diagnostic criteria or severity of IAD to assess impulsivity, executive function, and working memory between Internet addictive individuals and alcohol-dependent patients. Additionally, this study results are preliminary because of the small sample size. Further studies with larger sample sizes are needed to replicate these findings.

## Conflict of Interest Statement

The authors declare that the research was conducted in the absence of any commercial or financial relationships that could be construed as a potential conflict of interest.
